# Distribution and behavior of lipid droplets in hepatic cells analyzed by variations of citochemical technique and scanning electron microscopy

**DOI:** 10.1016/j.mex.2022.101769

**Published:** 2022-06-21

**Authors:** João Marcos de Lima-Faria, Lucas Nunes Guimarães, Victória Costa da Silva, Iara da Costa Souza, Marisa Narciso Fernandes, Diego Stéfani Teodoro Martinez, Simone Maria Teixeira de Sabóia-Morais

**Affiliations:** a Laboratory of Cellular Behavior, Institute of Biological Sciences, Federal University of Goiás, Brazil; b Dept. Physiological Science-CCBS, Federal University of São Carlos, Brazil; c Brazilian Nanotechnology National Laboratory (LNNano), Brazilian Center for Research in Energy and Materials (CNPEM), Campinas, São Paulo, Brazil

**Keywords:** Lipid, Osmium, Hematoxylin, Toluidine blue, Scanning electron microscopy, Backscattered electrons, Oxidative stress, Steatosis, Hepatotoxicity

## Abstract

Toxicity evaluations involve the analysis of multiple biomarkers. In this study, the liver, target organ analyzed by treatments with iron concentrations, indicated the accumulation of lipids as a response. Considering that the distribution of lipids in an organ is directly related to the induction of inflammatory processes by aquatic contaminants, this study proposes to carry out an integrative investigation of the behavior and the distribution of lipids in the liver tissue. Techniques of light and electron microscopy were performed in order to propose a new way of assessing and quantifying the distribution of lipid droplets, also presenting methodological alternatives that can be chosen by the reader according to the interests and resources available. Thus, it is assumed that the method begins with the fixation of the liver with Glutaraldehyde 2,5% in PBS 0,1 M and continues with post fixation with osmium tretoxide 1%, which marks lipids. For this proposition, two inclusion methodologies were performed to histological analyses in Historesin and ultrastructural analyses in EMBeed 812. For light microscopy (LM) analyses, cuts were obtained with 2,5 micrometers thickness, which were stained with (1) Mayers hematoxylin and (2) toluidine blue. The images obtained were processed in software Image J Fiji to evidence the lipid distribution in liver.

• Cytological reactions with osmium tetroxide constitute low complexity methods that allow the optimization of the localization, identification and quantification of lipid droplets in the liver tissue when analyzed under the conventional light microscope.
• Samples included in EMBeed 812 resin commonly used in Transmission Electron Microscopy can be analyzed by SEM-BEC, as complementary analyses for the detection of lipids.
• Using SEM-BEC and conventional light microscopy, it is possible to quantify the area occupied by lipid droplets using Image J Fiji software, as these are contrasted due to the reaction with osmium tetroxide.

## Graphical abstract

**Figure:**
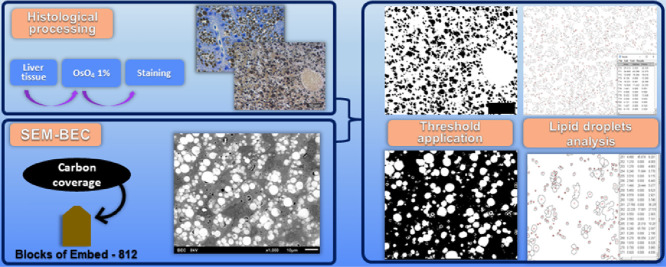

## Specifications table

**Table:** 

Subject Area:	Biochemistry, Genetics and Molecular Biology
More specific subject area:	*Risk assessment – Oxidative stress*
Method name:	*Evaluation of lipid distribution in osmium-fixed hepatic cells by light microscopy and scanning electron microscopy.*
Name and reference of original method:	A method for staining epoxy sections for light microscopy [8]
Resource availability:	*Image J Fiji*https://imagej.nih.gov/ij/download.html*and*https://imagej.net/Fiji/Downloads

## Method details

### Toxicity test around the world

To ensure adequate environment and human health conditions, it is necessary for freshwater quality to be constantly evaluated by toxicity tests. This allows us to assess risk, indicate the danger of contamination and suggest safe means of using substances. These tests are based on international guides whose technical standards indicate the use to various species, of which fish are the aquatic models of excellence for investigating toxic effects. Among the species indicated for the tests *Danio rerio* - with a 70% genome close to humans - and *Poecilia reticulata* - are interesting environmental biomonitors that have been widely endorsed.

Given the relevance of understanding the toxicity of substances and mixtures, researchers in the field seek biomarkers approach to understand the relationship between the complexity of the interaction of contaminants with the organism. Thus, in the extensive set of biomarkers, assessing oxidative stress involving processes of lipid peroxidation and accumulation of lipids in fish has ground because it reflects aspects of animal health related to the effects of food and water exposed to environmental contamination [1], [2], [3], [4], why lipid droplet formation is immediately related to the activation of defence mechanisms and reversal of oxidative stress [5,6]. In this sense, this methodological proposal brings: (1) an improvement in the lipid marking techniques with osmium, already described in previous works [7,8]; and (2) a new proposal for analyzing the organization of lipid droplets in fish liver, using light microscopy and elementary analysis interface of scanning electron microscopy.

In this sense, this work unites techniques of light microscopy and electron microscopy in order to offer possibilities to researchers according to their own reality, so that histological analyses allow a broader visualization of the tissue surface and a more precise observation of the relation between lipid droplets and cellular components.

### Methodology description

This study was approved by the Animal Research Ethics Committee of UFG (CEUA) under registration number 046/2017. The samples used in this work were collected in an experiment conducted by the research group of the Laboratory of Cell behavior with the use of females of *Poecilia reticulata* submitted to exposure to pesticides based on phosphomethylglycine. The animals were exposed to the contaminant for 21 days. Then, they were sacrificed for cooling and dissected in the supine position for liver extraction, which was divided into two fragments, fixed for 24 h in 2.5% glutaraldehyde solution diluted in 0.1 M sodium phosphate buffer.

The highlight of this work is the post-fixation for 2 h in a 1% osmium tetroxide solution, since the osmium has an affinity for lipid molecules, contrasting the phospholipids of the plasma membrane and the endomembrane system, as well as the lipid droplets in the cytoplasm. Both fixation and post-fixation were performed at 4 °C and the fragments were distributed according to the following methodologies.

#### Light microscopy analyses

Fragments of liver were dehydrated for 30 min in 70% alcohol and 30 min in 95% alcohol, followed by infiltration of glycol methacrylate (Historesin, Leica, Germany). The infiltration started in a 1:1 solution of historesin and absolute alcohol (v / v) for 2 h, then in a 2:1 solution of historesin and absolute alcohol (v / v) for 1 h and, finally, in pure historesin for another 2 h. After infiltration, the material was placed in histological molds and included in a mixture of historesin with benzoyl peroxide (50 mL/0.5 g), being polymerized in an oven at 60 °C for 48 h. After polymerization, the samples were taken to the ultramicrotome (Leica Ultracut UCT, Leica), where sections of 2.5 µm in thickness were obtained. The cuts were transferred to a glass slide with drops of water and placed to dry on a hot plate. The assembled blades proceeded to two different protocols, as follows.

Important: osmium precipitates in solvents such as alcohol and xylene, therefore, the sections show instability in osmium staining if the slide is assembled with Entellan® mounting medium. Our team tested other options, including those with montage medium glycerin-gelatine, but the cuts deformed, due to its hydrophilic nature. Therefore, the most suitable way for its chemical properties was using Entellan®. Thus, immediately before the analysis, the slides must be assembled, and the entire evaluation must be carried out within a maximum of 24 h because after this period, the reaction promoted by the interactions with the osmium starts a reverse reaction and the colour for being labile begins to get lost. Then, the marked tissue becomes transparent.

##### Osmium with Mayer's hematoxylin

**Fig. 1:**
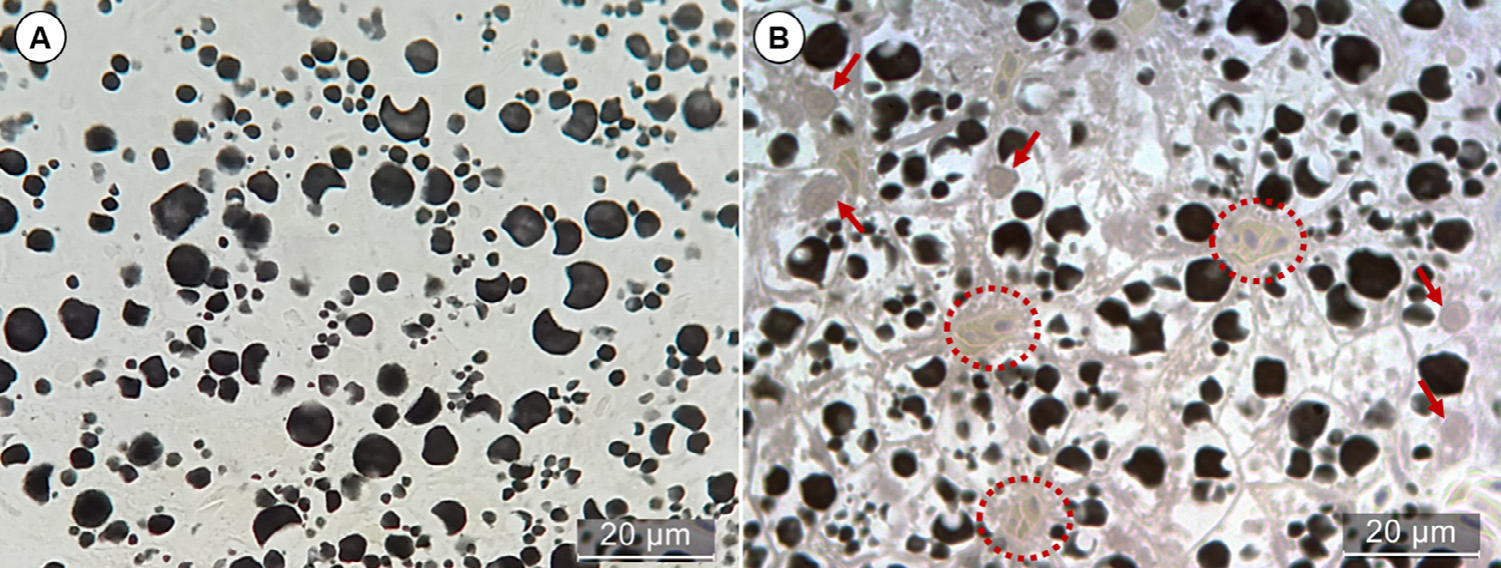
*Poecilia reticulata* liver tissue post-fixed with osmium before (A) and after (B) Mayer's hematoxylin staining. Without hematoxylin staining (A), it is not possible to locate the droplets in the cells, while in stained tissue (B) it is possible to visualize the nuclei (red arrows), as well as other structures such as blood vessels (dotted).

**Fig. 2:**
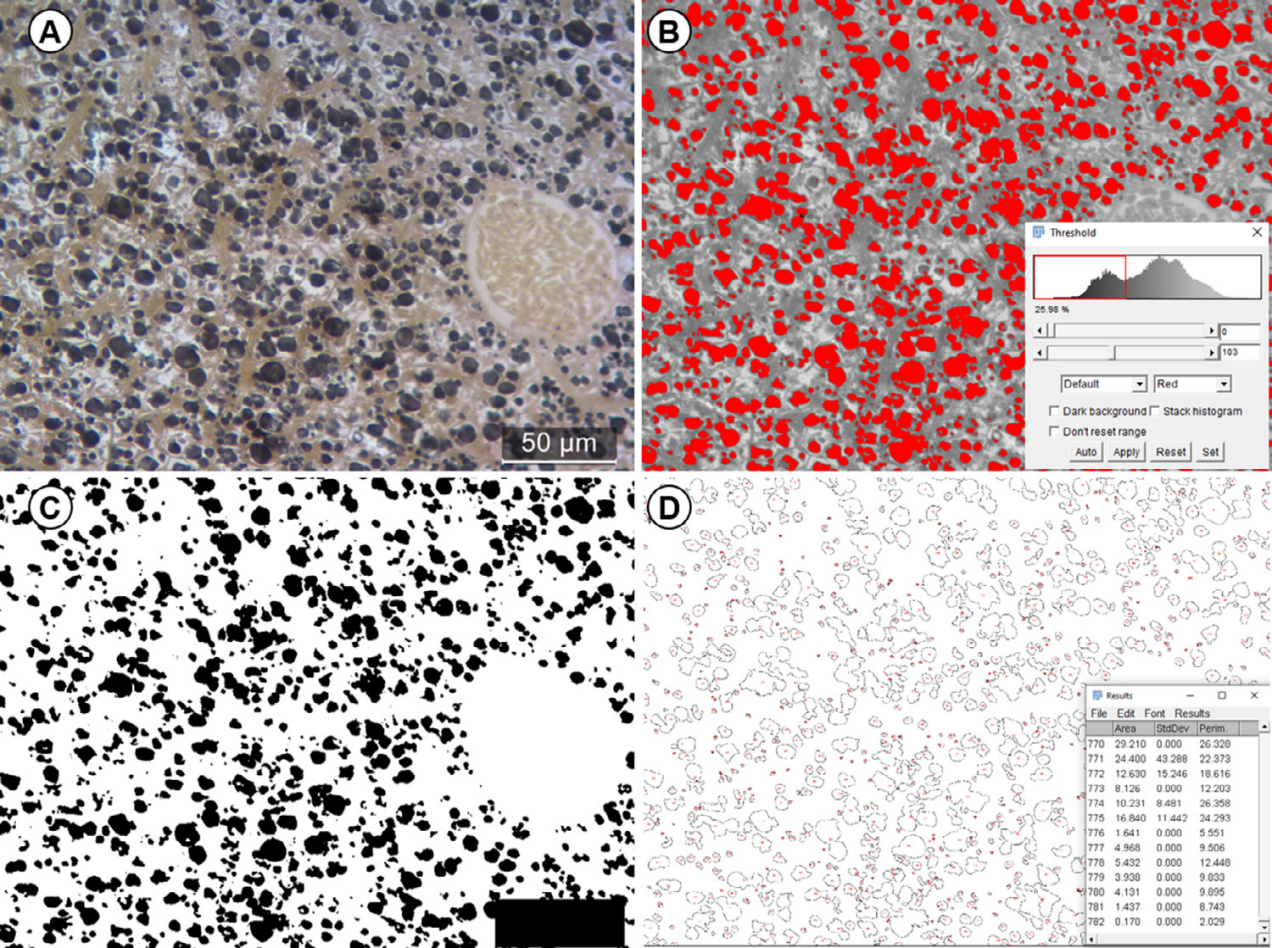
Application of the threshold technique in Image J Fiji software to visualize the organization and counting of lipid droplets in *Poecilia reticulata* liver sections stained with Mayer's hematoxylin. Original image without changes (A); Phase of delimitation and adjustment of coverage of lipid droplets, in this phase the range of tones that will mark only the droplets is defined, according to step 2 of Table 1 (B); Threshold applied, in this phase artifacts and scale bar are excluded, according to step 3 of Table 1 (C); Measurement of clusters and lipid droplets, in this phase the minimum size of the droplets to be measured is defined and the value of the scale bar region is excluded, according to step 4 of Table 1 (D).

For staining with Meyer's hematoxylin, the slides with sections were hydrated by immersion in distilled water for 10 min, proceeding to immersion in Mayer's hematoxylin for 35 min, then the slides were washed in running water for 5 min and dried in a drying oven. It is important to note that historesin is dissolved in alcohol, so Meyer's hematoxylin was the option chosen to stain the sections because it does not contain alcohol in its formulation, also because it is a progressive dye marking basophilic tissue compounds such as cell nuclei, membranes and fibers, as its cellular components have negative charge, interacting well with the alum that is the mordant in the dye solution. Thus, the staining with Mayer's hematoxylin allow a better assessment of cell morphology, on the other hand, it does not interact with the osmium and allows the perfect visualization of lipid droplets in the tissue and its intracellular disposition, as shown in Fig. 1. Because of the contrast generated with the labelling of lipids by osmium tetroxide, it is possible to apply the threshold technique using the Image J software, following the steps in Table 1, so that the analysis of contrasted particles allows to obtain a notion of the area occupied by lipid droplets in a wide region of the organ, since the technique works well in photomicrographs acquired with lenses as small as 20x (Fig. 2)

**Table 1:** Image processing Step-by-step to realise threshold and obtain data of area and quantity of lipid droplets on software Image J Fiji.

	Step	Activity
1	Open image and set scale	• (1) Draw a line on the scale bar of the image; (2) analyze, set scale; (3) enter the distance known as the scale bar size; (4) enter the unit of measure.
2	Apply the threshold to the image (Fig. 2B)	• (1) Access Image, digitalise and transform image in 8-bit; (2) Image threshold or CTRL+Shift+T; (2) set the interval in the histogram to mark only lipid droplets and apply the threshold. Note that some regions that are not of interest may be marked, they will be removed in the next step.
3	Remove unnecessary information (Fig. 2C)	• Using the rectangle tool, delete areas with unnecessary information (e.g. scale bar, texts, vessel regions, artefacts and imperfections).
4	Measuring lipid droplets (Fig. 2D)	• (1) Analyze particles; (2) set a minimum measurement size, to prevent the software from counting very small spots that can be waste or osmium traces that are not of interest, we suggest a minimum size of 0.15 µm^2^; (3) to see the labeled droplets, select Show, overlay or outlines; (4) Ok; (5) In the list of measurements remove the measurement relative to the space occupied by the scale bar and the items removed in step 3.
5	Check	• Check that there are no undesirable items marked and that as many lipid droplets as possible have been marked, if you want, change the minimum size and shape of the particles desired to measure.

##### Osmium with toluidine blue

Continuing the proposal to evaluate lipid droplets, this study presents the technique of post-fixed tissue with osmium stained with toluidine blue as an option to be used by the reader for a more detailed understanding of the relation between tissue structures, such as between cytoplasmic space, the different cell types and the with blood vessels.

In this staining method, immediately after drying the slides, the sections were covered with 1% toluidine blue solution at pH 5.2 and placed on a hot plate until a golden ring was formed on the dye drops - which takes approximately 1 min at 55 °C, after which, the slides were washed with distilled water to remove the excess dye. At this stage, it is important to observe whether the intensity of the staining is of interest to the user, because as toluidine blue and osmium combine, the staining can be too intense, especially in the medullary regions of the liver, if this occurs leave the slides on the heating plate for a shorter time.

**Fig. 3:**
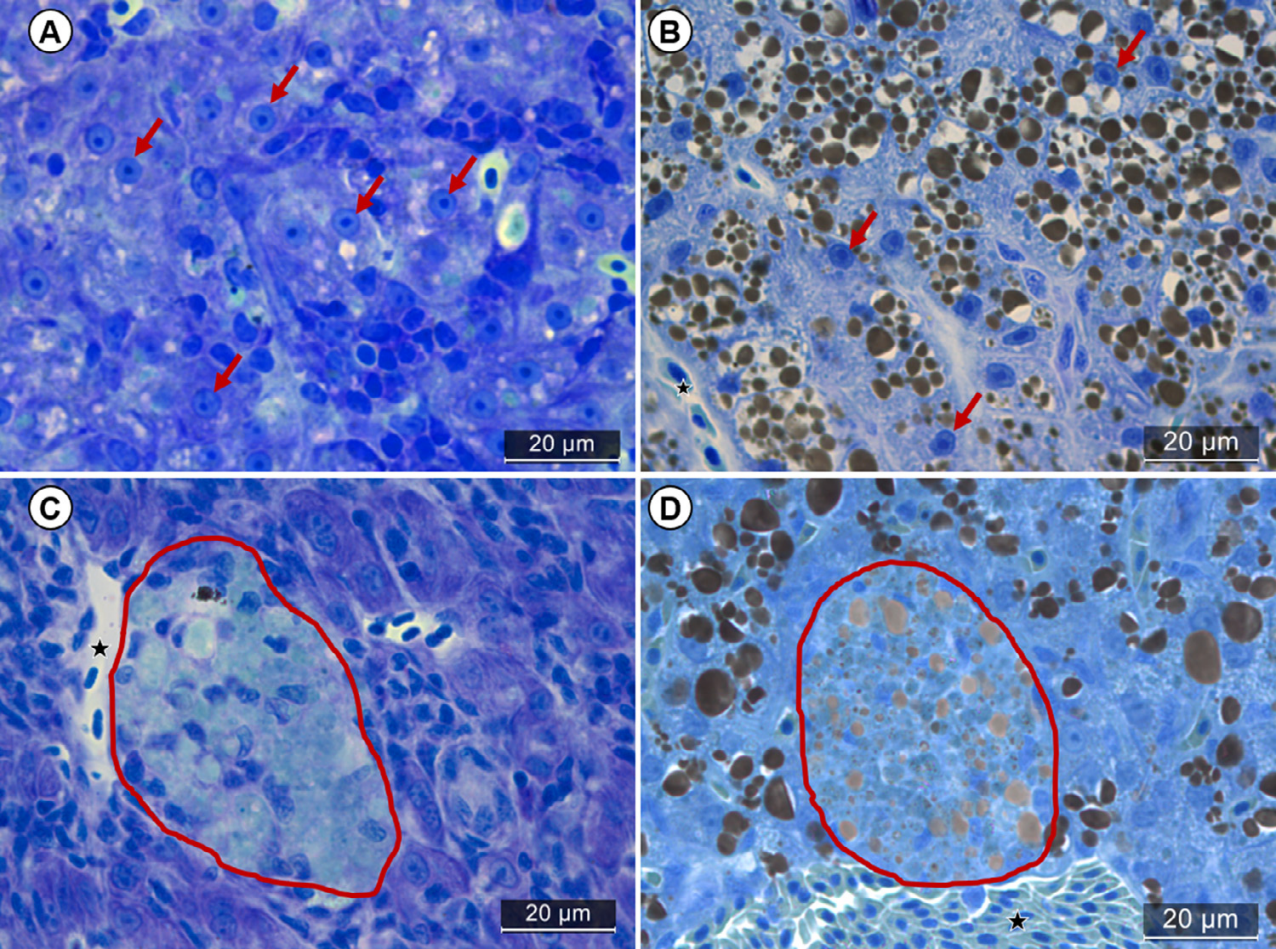
Toluidine blue staining of *Poecilia reticulata* liver tissue without osmium (A,C) and post-fixed with 1% osmium (B,D). Toluidine blue staining alone traditionally marks most of the structural components of cells, with greater intensity in nuclei (red arrows), and also marks melanomacrophage sites (red rounded) in lighter shades of blue and does not stain cytoplasm of erythrocytes with intensity. On the other hand, when toluidine blue staining is applied to post osmium-fixed tissue it is possible to note the increased delimitation of cells by the intensity of membrane staining (B,D). Because of the blue+osmium combination the red blood cells are highlighted in the vessels (star) and in the melanomacrophage sites it is possible to identify the areas of lipofuscin pigment arranged in light brown droplets in the sites (D).

Toluidine blue is widely used for staining samples embedded in historesin, as it recognizes carboxyl, sulphate and phosphate groups in tissues when at a neutral pH of 4.0. Thus, toluidine blue was chosen because it stains beyond the nucleus, the cytoplasm and cell membranes (Fig. 3). In this sense, the combination of toluidine blue with osmium highlights the membranes by the sum of the contrast of the dye with osmium (Fig. 3B and D), which allows us to analyze the distribution of lipid droplets and their respective organization in the cells, also highlighting structures that only the osmium staining would not highlight, such as melanomacrophage sites, exudates and leukocyte infiltrations (Fig. 3D). Furthermore, the combination of the techniques in historesin allows us to relate the arrangement of lipids with processes of inflammatory responses in relation to exposure to contaminants, such as the localization of lipid congested ducts, the formation of macro- and micro-stasis in hepatocytes (Fig. 3B), as well as the localization of lipofuscin at melanomacrophage sites (Fig. 3D).

#### Scanning electron microscopy analyses

The methodology described below was thought to take advantage of material destined for transmission electron microscopy to answer complementary questions about the behavior of lipids droplets in the liver of fish exposed to contaminants, with this, the material was optimized to obtain in the same sample ultrastructural information by TEM and elemental information by SEM. In this case, this methodology presents as an option to better visualize the relation between lipidic cells in the cytoplasm cells in a more precise way.

In this sense, the second liver fragment, after post-fixation with osmium tetroxide, followed the sample processing for transmission electron microscopy. The sample was dehydrated in acetone 30–100% and then infiltrated in: (1) 1:1 (v/v) solution of epoxy resin (Embed 812, EMS) and acetone absolute for 24 h, (2) 2:1 (v/v) solution of epoxy resin and acetone absolute for another 24 h, and (3) pure epoxy resin for 24 h. The infiltrated material was placed in BEEM® capsules and polymerized in an oven at 60 °C for 48 h. After polymerization, the blocks obtained were trimmed and taken to the ultramicrotome (Leica Ultracut UCT, Leica), where they were thinned until the exposure of the tissue in the resin block. Other sections were obtained for transmission electron microscopy that complemented the other work of the team, which shows the optimization of the material in the research.

The roughened blocks were positioned in stubs vertically, so that the surface with the sample was upwards, then the stubs with the samples were taken to the university's multi-users high-resolution electron microscopy laboratory (LabMic) for coating with an evaporation carbon coating system (JEE-420, JEOL). The stubs with the blocks were taken to the scanning electron microscope JEOL JSM-6610 in capture mode of backscattered electrons.

##### Backscattered electron scanning electron microscopy (SEM-BEC): imaging

In the field of natural sciences, it is conventional to use images obtained by secondary electrons, these electrons have energy below 50 eV and provide topographic information when interacting with the valence layers of atoms in scanning electron microscopy (Fig. 3A). However, seeking to identify the lipids in the samples, the research team sought to associate elemental analysis techniques to obtain accurate information, in this sense, analyses were performed by capture of backscattered electrons.

The backscattered electrons interact with the nuclei of the sample atoms, so that more electrons are detected as the atomic number of the element increases. Osmium has a high atomic number (76), so by backscattered electron analysis the regions with osmium will be brighter. As osmium has an affinity for lipids, using this technique the lipid droplets will also be brighter, which is confirmed in Fig. 3B.

##### Backscattered electron scanning electron microscopy (SEM-BEC): image processing with Image J Fiji

The images obtained by backscattered electrons (Fig. 3B) can identify the composition of samples by the contrast of the images. Seeking to take advantage of the high contrast of the lipid droplets in images, in this work an analysis is proposed using the open-source software Image J Fiji [9]. Table 1 lists the steps to be followed in the software.

**Fig. 4:**
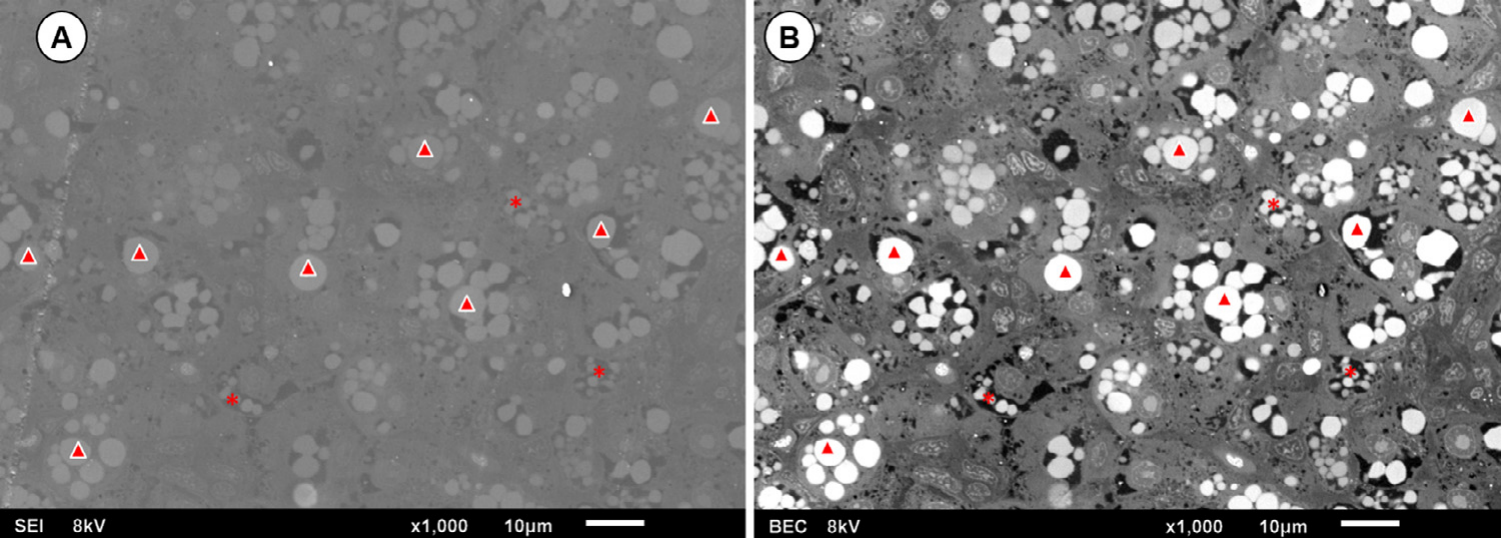
Scanning electron microscopy of the liver tissue of *Poecila reticulata* with images obtained by secondary electron detection (A) and backscattered electron detection (B). Secondary electron detection (A) provides images of the topography of the sample, so in this case it is an image with low contrast between the elements. On the other hand, backscattered electron detection (B) provides elementary information, with brightness proportional to the atomic number of the materials. Because of the osmium affinity with lipids and its high atomic number in relation to the other elements in the sample, it is possible to observe that both macro steatosis (triangles) and micro steatosis (asterisks) are highlighted with high brightness, allowing an analysis with greater definition of the distribution of lipids in the tissue.

**Fig. 5:**
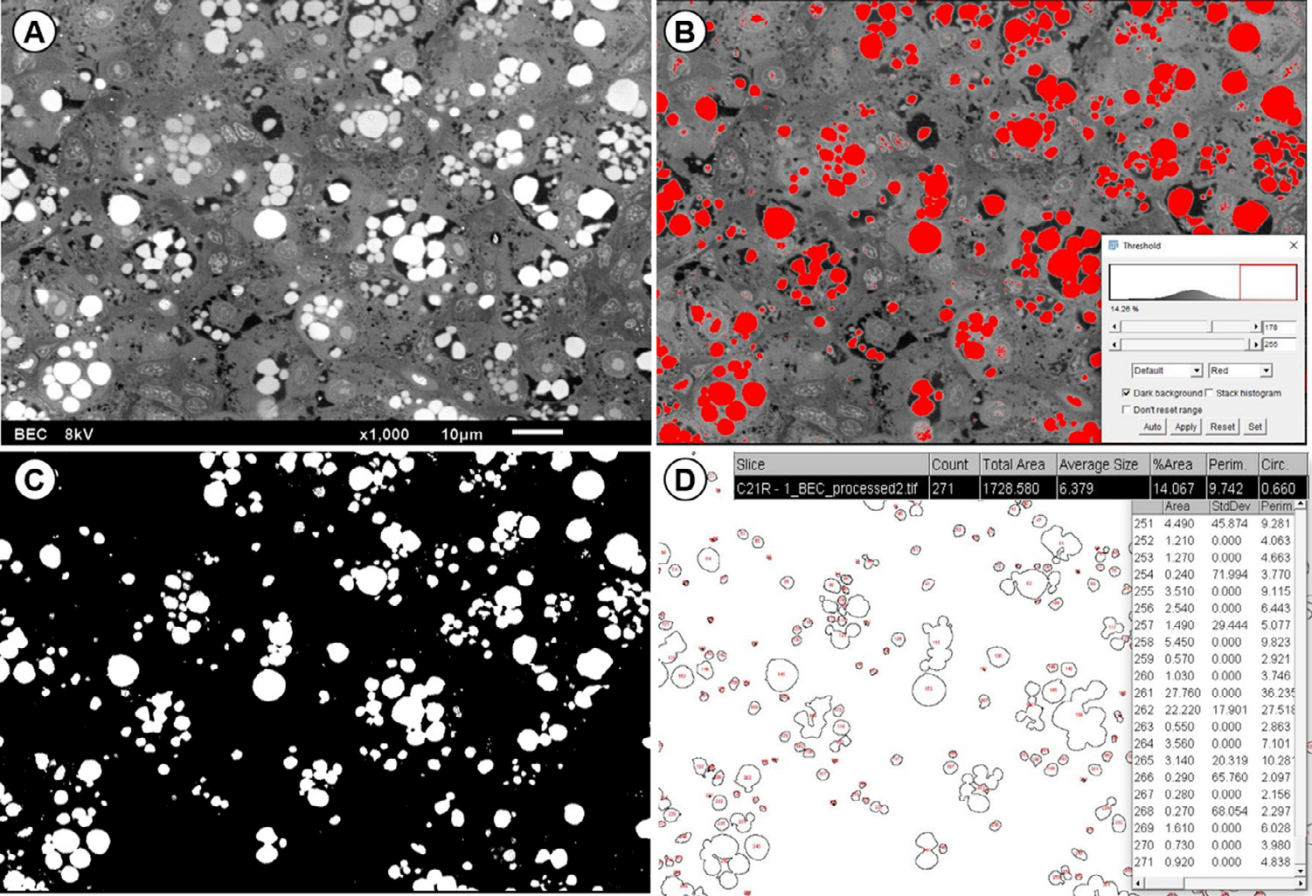
SEM-BEC image processing in Image J Fiji software. The original image is opened in the program and the scale is set (A); after the scale is set, the threshold area is defined to identify only the lipid droplets (B); the areas with artifacts and objects that are not of interest are cleaned and the threshold is applied (C); the minimum detection size is set in the particle analysis and measurements of the quantity and area of the lipid droplets are performed (D).

The analysis using Image J Fiji shows the area of the droplets, as well as allow us to evaluate the distribution of lipids and diagnose the formation of micro and macro steatosis (Fig. 4). Thus, in conjunction with the histological analyses described here, evaluation by SEM-BEC is of high complementary potential, since it is possible to observe and quantify micro and macro steatosis in hepatocytes, allowing to suppose levels of damage caused by the accumulation of lipids in the organs (Fig. 5).

## Methods validation

**Fig. 6:**
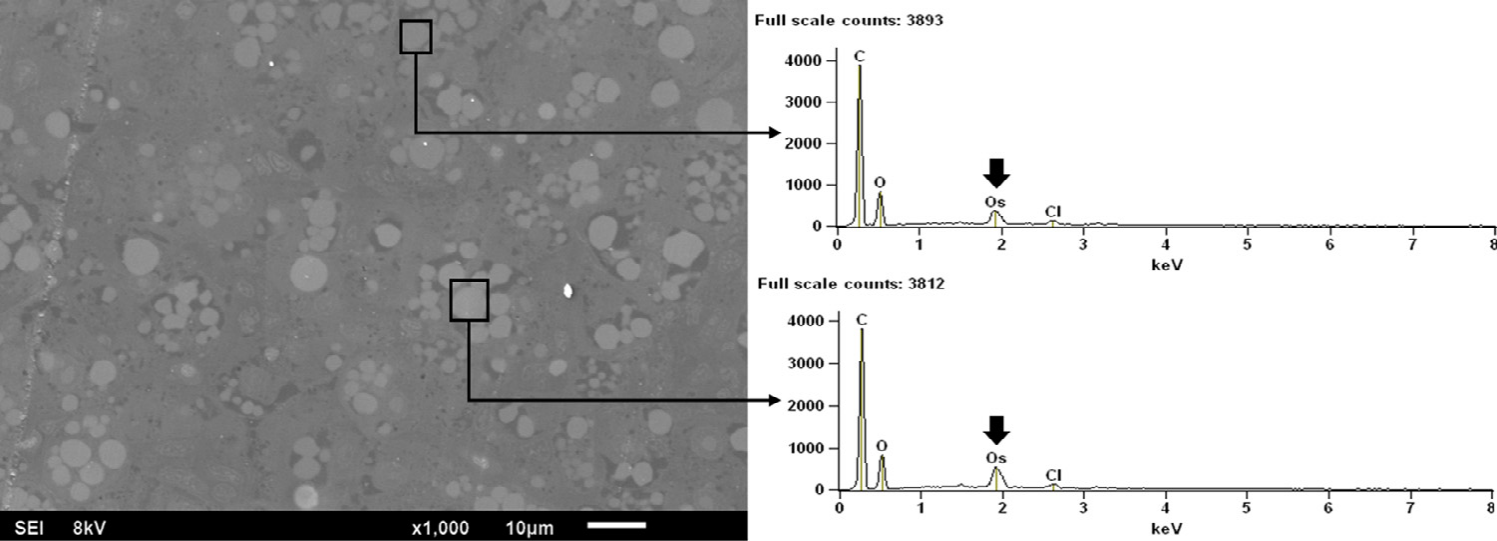
Elemental analysis by energy dispersive Xray spectroscopy (EDS) in liver tissue of *Poecilia reticulata*. The spectrums correspond to highlighted zones in electronmicrography and it is possible to note the osmium presence (bulk arrow) in lipid droplets.

For the light microscopy evaluations Fig. 1B shows the difference of the tissue stained with Mayer's hematoxylin when compared to Fig. 1A without hematoxylin. Similarly, toluidine blue staining is validated by comparing the images with the traditional staining method (Fig. 3A and C) and those with osmium-fixed material (Fig. 3B and D). The SEM-BEC technique was validated by performing elemental analysis by energy-dispersive X-ray spectroscopy (EDS), as shown in Fig. 6, confirming the presence of osmium in regions of intense bright staining the lipid droplets.

## Declaration of Competing Interest

The authors declare that they have no known competing financial interests or personal relationships that could have appeared to influence the work reported in this paper.

## Data Availability

Data will be made available on request.
